# Social media video analysis methodology for sarin exposure

**DOI:** 10.1080/20961790.2020.1825061

**Published:** 2020-11-05

**Authors:** Sadik Toprak, Emine Yilmaz Can, Bulent Altinsoy, John Hart, Zekeriya Dogan, Mustafa Ozcetin

**Affiliations:** a Department of Forensic Medicine, Istanbul University, Istanbul, Turkey; b Department of Medical Pharmacology, Bulent Ecevit University, Zonguldak, Turkey; c Department of Pulmonary Medicine, Bulent Ecevit University, Zonguldak, Turkey; d James Martin Center for Nonproliferation Studies, Monterey, CA, USA; e Department of Civil Engineering, Bulent Ecevit University, Zonguldak, Turkey; f Department of Pediatrics, Istanbul University, Istanbul, Turkey

**Keywords:** Forensic sciences, social media, sarin, scale, questionnaire, chemical weapon, nerve agent, YouTube

## Abstract

As social media becomes increasingly ubiquitous, many events are recorded and released on social media platforms, including chemical weapon attacks. We develop an objective tool in order to evaluate brief and unstructured social media videos for analysing sarin exposure from a civilian medical pathology perspective. We developed and validated this new questionnaire using a standardized procedure that includes content domain specification, item pool generation, content validity evaluation, a pilot study, and assessment of reliability and validity. In total, 51 sarin attacks and 48 matched videos were analysed. Cronbach’s α for all 20 items was 0.75, which indicates adequate internal reliability. The test–retest reliability was 0.96, which indicates good internal reliability. The inter-observer intraclass correlation coefficient was 0.97. After verifying sampling adequacy with the Kaiser–Meyer–Olkin measure and the factorability of the items with Barlett’s test of sphericity, a factor analysis was performed. According to the principal axis factoring, a six-factor solution explained 51.86% of the total variance. The receiver-operating characteristic curve analysis showed that the Video Score Questionnaire has a sensitivity of 0.817, a specificity of 0.478, and an efficiency of 65.3. Therefore, the Video Score Questionnaire is reliable and valid for evaluating sarin attacks from brief and unstructured social media videos.

**Key points**
- Chemical weapons are still used as a method of warfare.
- Social media videos are an important source of information.
- We developed a validated scale which can analyse sarin exposure in short and unstructured videos.

## Introduction

Chemical weapons attacks have occurred in Syria since at least 2013 and more recently in Iraq. The Organisation for the Prohibition of Chemical Weapons (OPCW), the body that implements the 1993 Chemical Weapons Convention (CWC) and the OPCW-United Nations (UN) Joint Investigative Mechanism (JIM) in Syria confirmed that the organophosphorus nerve agent sarin was used in Syria [1]. In 2020, an OPCW Investigation and Identification Team (IIT), established in June 2018 to attribute responsibility for chemical weapon attacks in Syria, issued its first report [2].

In February 2017, Kim Jong-Nam, the half-brother of North Korea’s leader Kim Jong-Un, was assassinated inside the Kuala Lumpur airport with an agent understood to be the nerve agent ethyl *N*-2-diisopropylaminoethyl methylphosphonothiolate (VX). Malaysian authorities placed two women on trial for the act in late 2017, and an autopsy reportedly identified VX in ocular and facial swabs [3].

As social media becomes increasingly ubiquitous, numerous events are recorded by mobile devices and released on open-source platforms. Social media videos have been used in various scientific fields, including for the diagnosis of medical conditions such as autism and accidental injuries [4–6].

Social media videos have been used by the UN and other international institutions (e.g. OPCW) as evidence of chemical weapon use in the ongoing civil war in Syria [7, 8]. Social media videos represent one of the most important information sources available. Such videos have also been used to support chemical weapon exposure evaluations in the medical literature [9].

No valid screening instrument from the field of civilian medical pathology appears to exist specifically for the detection of chemical weapon exposure in social media videos [10]. In particular, the physiological effects of sarin can be analysed in social media videos using a questionnaire. Indeed, medical researchers routinely undertake to create reliable and valid tests and questionnaires in order to enhance the accuracy of their assessment and evaluations [11]. Such an approach is distinct from corresponding efforts undertaken in chemical weapons arms control and intelligence assessment contexts within the defence sector.

Sarin has immediate direct effects on the nervous system. Depending on the dose, species and route of administration, sarin can cause death within minutes [12]. Mild to moderate exposure to sarin vapour may result in local effects (i.e. miosis, blurred vision, and hypersecretions). Bronchoconstriction and respiratory distress may appear before pronounced symptoms involving the gastrointestinal tract develop. Small to moderate dermal exposure to liquid nerve agent produces increased sweating and muscular fasciculations; nausea, vomiting, and diarrhoea. Generalized weakness may be more marked. Large-dose exposures rapidly produce loss of consciousness, convulsions, flaccid muscle paralysis, and respiratory and circulatory failure and death [9, 12, 13].

The aim of this research is to develop an objective tool (i.e. a questionnaire) to evaluate short, unstructured social media videos for assessing sarin exposure from a civilian medical pathology perspective.

## Materials and methods

We developed an objective tool to assess exposure to chemical weapons in humans using short, unstructured, social media videos. The scale was designed to be used as a diagnostic tool. We therefore developed and tested a questionnaire (called the “Video Score”, Supplementary Figure S1) as the first step. Next, the questionnaire was tested using a group of conventional weapon attack videos in order to determine its diagnostic value.

Our sarin exposure video analysis questionnaire included 20 primary questions probing survival, neurological, autonomic nervous system, eye, pulmonary, circulatory, gastro-intestinal and therapeutic/decontamination aspects observed in the video footage. As a diagnostic instrument, the Video Score items are dichotomized to indicate the presence (=1) or absence (=0) of each of the symptoms. The scale language was English.

Data were collected in December 2017. The Ethics Committee of Bulent Ecevit University approved the study protocol (ethics code 33479383/46).

### Study participants

A random, computer-generated number was used to select each participant from the medical faculty hospital. Thirty medical doctors and interns were selected among English-speaking medical staff from a university hospital. One individual declined to participate. All participants provided written informed consent prior to participation. The 29 participants viewed and rated each original and matched video.

### Sarin attack videos

A sarin attack took place on 21 August 2013 in the eastern part of Damascus (Ghouta) and killed ∼1 400 civilians and severely affected thousands more [8]. A UN team operating under the authority of the UN Secretary-General confirmed this attack [14]. Rosman et al. [9] published an article related to this attack and analysed 67 social media videos according to a delineation of the clinical presentation of casualties. When data were collected in September 2017, 51 videos were available and recorded from open-source platform. These 51 videos were determined to be original videos documenting a sarin attack.

### Matching attack videos

In September 2017, we searched YouTube (http://www.youtube.com) using the keywords “Syria”, “conflict”, “attack”, and “hospital”. Matching videos were collected from conventional weapon attacks. Four matching criteria were determined: language (Arabic), duration, number of injured people, and crime scene. In total, 51 videos were initially collected, with a final total of 48 videos after duplications were removed. The full list of videos is provided in Supporting Information.

### Scale development

#### Content domain specification

First, a review of the literature was performed to discover information related to sarin. At this step, dimensions of sarin exposure symptoms in humans were defined, namely, clinical findings and treatment.

#### Item pool generation

All clinical findings and treatment options were included according to the literature review regarding the physiological effects of sarin.

#### Content validity evaluation

A professional focus group that was not involved in the construction process of the scale considered the clinical importance of the items prior to their removal as well as their content validity. For content validity, a panel of experts that included a pharmacologist, a forensic pathologist, a pulmonologist, and a consultant in emergency medicine reviewed and revised the items. These experts assessed the appropriateness of the items for their respective constructs [15].

Four medical doctors utilized face validity to review grammar, syntax, organization and appropriateness of the Video Score. This group was convened because face validity requires that the measure appear relevant to the construct to an innocent bystander [16].

#### Pilot study

A pilot study was performed by five medical doctors. A professional focus group reduced the number of items in two cases: they removed items that showed redundancy of measurement by a high correlation with another item (*r* > 0.80), and they removed items if the correlation coefficient between each item and the total score, excluding an item, was very low when compared with that of other items.

#### Reliability assessment

For internal consistency, the homogeneity of the items was evaluated using Cronbach’s coefficient. A coefficient of 0.7 or higher is preferred for a questionnaire to be internally consistent [17]. However, as our scale analysed different aspects of a complex clinical phenomenon (i.e. sarin exposure), we also performed test–retest reliability [18]. An intraclass correlation coefficient (ICC) was calculated; a score above 0.7 is considered a good indication of the questionnaire’s test–retest reliability [17]. The interval between the test–retest was 30 days. Lastly, the inter-observer reliability was assessed by ICC based on two-way random effects models.

A factor analysis (i.e. principal axis factoring with varimax rotation) was performed in order to confirm that the selected items were combined into relevant symptom clusters. *A priori* criterion for domain identification was eigenvalues >1.0.

#### Validity assessment

Receiver-operating characteristics were calculated to determine the sensitivity, specificity, positive and negative predictive values (PPV and NPV) and efficiency of each cut-off score [19]. Sensitivity was defined as true positives/(true positives + false negatives). Specificity was defined as true negatives/(true negatives + false positives). PPV and NPV were defined as follows: true positives/total number of test positives and true negatives/total number of test negatives, respectively. The efficiency of the test was defined as follows: (true positives + true negatives)/total number of subjects.

Statistical analyses were conducted using SPSS 21.0 software (IBM Corp., Armonk, NY, USA; licenced to Istanbul University).

## Results

The final questionnaire consists of 20 items and descriptives, as listed in Table 1.

**Table 1.** Descriptive values of sarin attack (*n =* 1 402) and control video (*n =* 1 316) scores obtained from the Video Score Questionnaire.

Symptoms	Sarin attack videos		Control videos		Total (*N =* 2 718)	
	Mean scores (±SD)	*n* (%)	Mean scores (±SD)	*n* (%)	Mean scores (±SD)	*n* (%)
Coma	0.40 ± 0.49	561 (40.0)	0.23 ± 0.41	299 (22.7)	0.32 ± 0.46	860 (31.6)
Loss of consciousness/unconsciousness	0.76 ± 0.42	1 061 (75.7)	0.50 ± 0.50	652 (49.5)	0.63 ± 0.48	1 713 (63.0)
Loss of muscle tone	0.44 ± 0.42	610 (43.5)	0.27 ± 0.50	353 (26.8)	0.35 ± 0.48	963 (35.4)
Muscle weakness (flaccid or spastic muscle paralysis)	0.26 ± 0.44	368 (26.2)	0.12 ± 0.32	158 (12.0)	0.19 ± 0.39	526 (19.4)
Circulatory failure	0.16 ± 0.36	223 (15.9)	0.10 ± 0.29	130 (9.9)	0.13 ± 0.33	353 (13.0)
Dyspnoea	0.54 ± 0.49	761 (54.3)	0.28 ± 0.45	374 (28.4)	0.42 ± 0.49	1 135 (41.8)
Respiratory failure/arrest	0.42 ± 0.49	593 (42.3)	0.26 ± 0.43	336 (25.5)	0.34 ± 0.47	929 (34.2)
O_2_ therapy	0.50 ± 0.50	700 (49.9)	0.36 ± 0.48	471 (35.8)	0.43 ± 0.49	1 171 (43.1)
Bewilderment/confusion	0.40 ± 0.49	564 (40.2)	0.31 ± 0.46	412 (31.3)	0.36 ± 0.48	976 (35.9)
Excessive salivation	0.20 ± 0.39	279 (19.9)	0.11 ± 0.31	148 (11.2)	0.16 ± 0.36	427 (15.7)
Cough	0.04 ± 0.20	62 (4.4)	0.01 ± 0.11	16 (1.2)	0.01 ± 0.16	78 (2.9)
Bronchospasm	0.17 ± 0.37	234 (16.7)	0.07 ± 0.24	87 (6.6)	0.12 ± 0.32	321 (11.8)
Nausea/vomiting	0.03 ± 0.16	37 (2.6)	0.02 ± 0.12	20 (1.5)	0.02 ± 0.14	57 (2.1)
Giddiness	0.10 ± 0.29	137 (9.8)	0.06 ± 0.23	74 (5.6)	0.08 ± 0.26	211 (7.8)
Headache	0.03 ± 0.17	43 (3.1)	0.02 ± 0.12	74 (5.6)	0.02 ± 0.15	211 (7.8)
Muscle fasciculations	0.27 ± 0.44	377 (26.9)	0.14 ± 0.34	179 (13.6)	0.20 ± 0.40	556 (20.5)
Convulsion	0.22 ± 0.41	315 (22.5)	0.08 ± 0.27	106 (8.1)	0.15 ± 0.36	421 (15.5)
Decontamination	0.15 ± 0.35	207 (14.8)	0.11 ± 0.30	140 (10.6)	0.13 ± 0.33	347 (12.8)
Diaphoresis	0.23 ± 0.39	325 (23.2)	0.11 ± 0.31	147 (11.2)	0.17 ± 0.36	472 (17.4)
Lacrimation	0.18 ± 0.38	253 (18.0)	0.10 ± 0.30	131 (10.0)	0.14 ± 0.34	34 (14.1)
**Total score**	**5.49 ± 3.19**		**3.23 ± 2.82**		**4.40 ± 3.22**	

### Internal reliability

The overall Cronbach’s α value for all 20 items was 0.75 using the whole dataset. This value (>0.7) suggests that the questionnaire has adequate internal reliability [20]. The test–retest reliability correlation of the Video Score exceeded the minimum acceptable correlation (interclass correlation: 0.96, CI: 0.94–0.97). The inter-observer ICC was 0.97 (CI: 0.96–0.98).

### Factor analysis

In the dimensionality analysis, the Kaiser–Meyer–Olkin measure verified the sampling adequacy KMO = 0.79), which is well above the acceptable limit of 0.5 [21]. The Barlett’s Test of Sphericity was significant (*P* < 0.01) and supported the factorability of the items.

A principal axis factoring with varimax rotation was conducted on the 20 items (Table 2). The six-factor solution accounts for 51.79% of the variance.

**Table 2.** Principal axis factoring analysis suggesting the six-factor solution and the total variance explained 51.79%.

Factor	Symptoms	Eigenvalues	% of variance
1	Coma Loss of consciousness/ unconsciousness Loss of muscle tone Muscle weakness (flaccid or spastic muscle paralysis) Circulatory failure	3.80	19.00
2	Dyspnoea Respiratory failure/arrest O_2_ therapy	1.67	8.35
3	Bewilderment/confusion Excessive salivation Cough Bronchospasm Nausea/vomiting	1.43	7.15
4	Giddiness Headache	1.34	6.73
5	Muscle fasciculations Convulsion Decontamination	1.09	5.47
6	Diaphoresis Lacrimation	1.01	5.09

**Figure 1.**
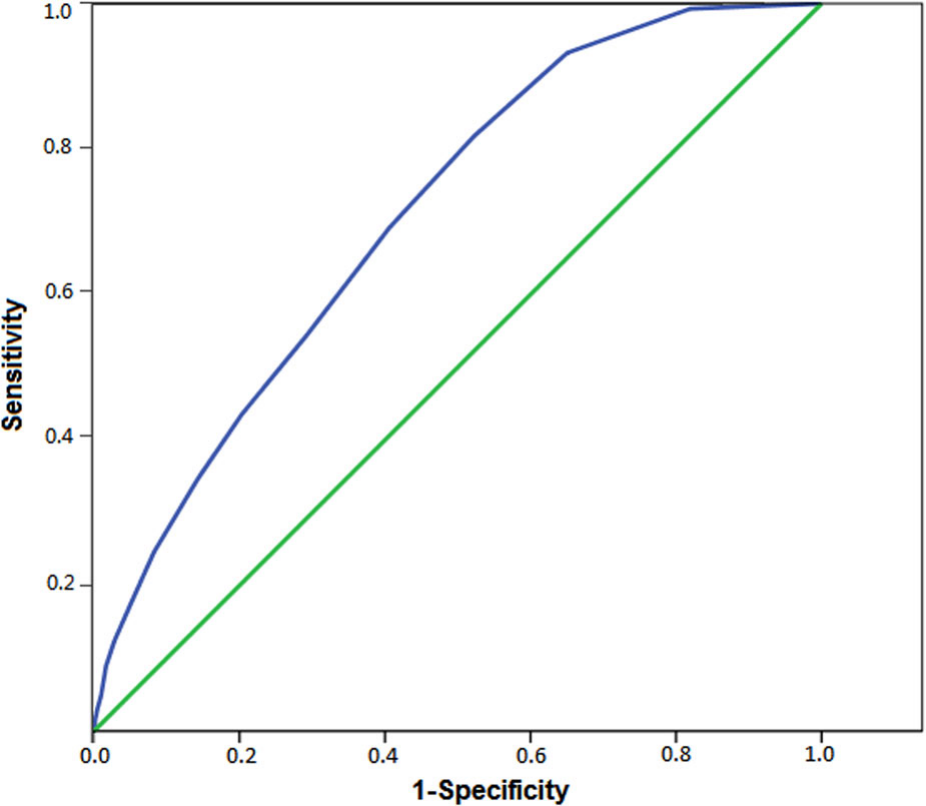
The receiver-operating characteristic curve for the Video Score. The area under curve is 0.71 (*P* < 0.01).

Better diagnostic scales exhibit curves that more closely approach the upper left corner (0.1). The area under the curve (AUC) is an index of the goodness of the diagnostic scale. The perfect scale has an AUC of 1.0 [22]. The AUC for the Video Score (Figure 1) is 0.71 (*P* < 0.01), which is considered to be “fair”.

Table 3 shows the results from the ROC analysis. The most appropriate cut-off point of the Video Score with a sensitivity of 0.817, a specificity of 0.478, and an efficiency of 65.3% was 2.5. A score >2.5 was selected as the optimal cut-off point to screen for sarin exposure as the highest total value of sensitivity and specificity reached.

**Table 3.** Receiver-operating characteristic results for all cases and each cut-off value.

Video Score cut-off	Sensitivity (%)	Specificity (%)	PPV (%)	NPV (%)	Efficiency (%)
>0.5	99.3	18.1	56.4	96.0	59.9
>1.5	93.2	35.0	60.4	82.9	65.0
>2.5	81.7	47.8	62.5	71.1	65.3
>3.5	69.3	59.3	64.5	64.5	64.4
>4.5	53.9	71.2	66.6	59.2	62.2
>5.5	43.5	79.7	69.6	57.0	61.0
>6.5	34.8	85.6	72.1	55.2	59.4
>7.5	24.7	91.7	76.0	53.3	57.1
>8.5	17.8	94.8	78.5	52.0	55.0
>9.5	12.4	97.2	82.5	51.0	53.4
>10.5	9.0	98.3	85.1	50.4	52.2
>11.5	4.9	99.0	84.1	49.4	50.4
>12.5	3.0	99.5	87.5	49.1	47.9
>13.5	1.5	99.8	91.3	48.8	49.1
>14.5	0.5	99.8	77.8	48.5	48.6
>15.5	0.3	99.9	80.0	48.5	48.5
>16.5	0.1	99.9	66.7	48.4	48.4
>18.0	0	100	–	48.4	48.4

PPV: positive predictive value; NPV: negative predictive value.

## Discussion

The results suggest that the Video Score can serve as a useful tool for screening sarin exposure in brief and unstructured social media videos. The optimum cut-off value is >2.5 to detect sarin exposure when compared with conventional weapon attack videos.

This study has strengths and limitations. A total of 29 participants took part in the study, and 51 sarin attacks and 48 matched videos were analysed. To our knowledge, this is the first study to analyse social media videos in order to develop an objective diagnostic tool from a civilian medical pathology perspective. However, our data are limited to only a single attack and a single country. It should also be noted that the AUC of our tool is only considered to be fair (AUC = 0.71) (Figure 1). In addition, the Video Score has limited sensitivity and specificity values: 81.7% and 47.8%, respectively (Table 3).

The reliability of the Video Score was analysed with Cronbach’s α, the test–retest reliability and an inter-rater reliability test. The results of all these tests were acceptable.

Sarin attack videos had higher positive values than matched videos for each item; also, average scores were 5.49 ± 3.19 and 3.23 ± 2.82, respectively (Table 1). According to the factor analysis, Factor 1 had the highest factor loading. Factor 1 can be considered a survival factor because it contains items related to torment, coma, loss of consciousness/unconsciousness, loss of muscle tone, muscle weakness and circulatory failure (Table 2). Although these symptoms are not unique to sarin exposure, they are typical symptoms of sarin exposure [23].

Respiratory symptoms aggregated in Factor 2 included dyspnoea, respiratory failure/arrest and O_2_ therapy (Table 2). Respiratory symptoms are one of the main symptoms of sarin exposure [12]. However, these symptoms can be seen in connection with other serious traumas, including conventional weapon attacks.

Factor 3 contains mixed symptoms associated with the central nervous (e.g. bewilderment/confusion, excessive salivation), gastro-intestinal (e.g. nausea/vomiting), and pulmonary (e.g. coughing, bronchospasm) systems. The most prominent symptoms in Factor 3 are pulmonary symptoms (bronchospasm in 16.7% of the sarin exposure group and in 6.6% of the matching group [6.6%] and coughing in 4.4% of the sarin exposure group and in 1.2% of the matching group).

Factor 4 represents impacts to the central nervous system, where both giddiness and headache are apparent in various trauma situations.

Muscle fasciculations, convulsion and decontamination constituted Factor 5 (Table 2). Muscle fasciculations and convulsion are frequent symptoms of sarin exposure. The third item, decontamination, is expected to be seen much more in sarin exposure videos; however, this was not the case. On average, decontamination was detected in 14.8% of sarin exposure videos and in 10.6% of the conventional weapon attacks. Hence, one can assume that decontamination facilities/activities were less indistinguishable than expected in our sample.

Factor 6 represents autonomic activity. Factor 6 contains two symptoms (i.e. diaphoresis and lacrimation), which are both symptoms of sarin toxicity. Table 1 shows that diaphoresis and lacrimation are more common in sarin exposure than conventional weapon attacks. However, these two symptoms can be seen in any other conditions that increase autonomic activity, such as a trauma resulting from a conventional weapon injury.

The optimum cut-off point was calculated to be 2.5 according to the receiver-operating characteristic (Table 3). The diagnostic sensitivity is higher than specificity at this point: 81.7% and 47.8%, respectively. Hence, the Video Score is more capable at detecting true positives than true negatives. Sensitivity and specificity values are limited because they cannot assist decision makers in making estimates of the probability of sarin attack from an individual video. To fill this need, predictive values were calculated [24]. Positive and negative predictive values describe a video’s probability of being a sarin attack once the results of the Video Score scale are known. Positive and negative predictive values were similar at this cut-off value: 62.5% and 71.1%, respectively. However, since the predictive values are strongly correlated with prevalence values, choosing videos to construct the Video Score scale is very important. In our sample, the prevalence of positive videos was 51.6% (1 402/2 718), and this value can be assumed as balanced. If, however, the Video Score scale applied a group of videos that contained a low percentage of sarin attacks, its positive predictive value would be lower than desired or expected.

In conclusion, we demonstrate the feasibility of applying a new and distinct (quantitative and qualitative) questionnaire in order to determine sarin exposure from a short, unstructured social media-video using a civilian medical pathology perspective.

## Supplementary Material

Supplemental Material
