# Supplementary material for: Social media video analysis methodology for sarin exposure
Source: Forensic Sci Res. 2020 Nov 5;7(2):279–84. doi: 10.1080/20961790.2020.1825061 (PMC9246001; doi:10.1080/20961790.2020.1825061)
Supplement: Supplemental Material [file TFSR_A_1825061_SM6633.zip › Supporting Information.pdf]

Supporting Information

Details on the 99 YouTube videos. Forty-eight match videos and 51 original videos samples were collected in total.

No	Sarin attack videos	Control videos
1	https://www.youtube.com/watch?v=KAD7Aw3VWl0	https://www.youtube.com/watch?v=CDtDMnzDa_w
2	https://www.youtube.com/watch?v=VKokjNNUdjc&list=PLPC0Udeof3T4NORTjYmPoNC Hn2vCByvYG	https://www.youtube.com/watch?v=14GXhdUftV0
3	https://www.youtube.com/watch?v=J24gD9JusMs&list=PLPC0Udeof3T4NORTjYmPoNCHn2vCByvYG	https://www.youtube.com/watch?v=sEs2HpX6UMY
4	https://www.youtube.com/watch?v=Y1vMd8mG9gE&list=PLPC0Udeof3T4NORTjYmPoNCHn2vCByvYG	https://www.youtube.com/watch?v=WpvuC-9_0kM
5	https://www.youtube.com/watch?v=rsZTf3DDkd4	https://www.youtube.com/watch?v=EYlwBvj03m0
6	https://www.youtube.com/watch?v=f20veT77OVk	https://www.youtube.com/watch?v=1QjHMiBT_7U
7	https://www.youtube.com/watch?v=oZn0_-TLc0U&list=PLPC0Udeof3T4NORTjYmPoNCHn2vCByvYG	https://www.youtube.com/watch?v=vvODhPoU7zU
8	www.youtube.com/watch?v=s0NAmopCLJE	https://www.youtube.com/watch?v=-jaYHnsuW7w
9	https://www.youtube.com/watch?v=7JT9RV3mZuA	https://www.youtube.com/watch?v=D2WeTLXATKc
10	https://www.youtube.com/watch?v=eql1X4Xs8jQ	https://www.youtube.com/watch?v=BKB3z0mQVQw
11	https://www.youtube.com/watch?v=UKLYZ8MDU7U&list=PLPC0Udeof3T4NORTjYmPoNCHn2vCByvYG	https://www.youtube.com/watch?v=L6vF2AgOCVE
12	https://www.youtube.com/watch?v=Uza8y7lcaGc&list=PLPC0Udeof3T4NORTjYmPoNCHn2vCByvYG	https://www.youtube.com/watch?v=uqbcZWYkAHY
13	https://www.youtube.com/watch?v=x6rGBqT8wz0	
14	https://www.youtube.com/watch?v=NmkN1CTxIlI	https://www.youtube.com/watch?v=jDXPVe-yCvA
15	https://www.youtube.com/watch?v=6O-G9HQru_o	
16	https://www.youtube.com/watch?v=9UU_uONafGE	https://www.youtube.com/watch?v=Be3bl6117w8
17	https://www.youtube.com/watch?v=7m5hHOz3d94	https://www.youtube.com/watch?v=Y3tj5sYxXlc
18	https://www.youtube.com/watch?v=4SbTdm0gIPk	https://www.youtube.com/watch?v=8qQwTo_9Cic
19	https://www.youtube.com/watch?v=un3wnAJ5vJs	
20	https://www.youtube.com/watch?v=AIi0cJZ2Yzk	https://www.youtube.com/watch?v=ev5IMiHUVso
21	https://www.youtube.com/watch?v=HNi1S9Cb-XI&list=PLPC0Udeof3T4NORTjYmPoNCHn2vCByvYG	https://www.youtube.com/watch?v=rvMAicLkpC0
22	https://www.youtube.com/watch?v=AqCDSq_BXKo&list=PLPC0Udeof3T4NORTjYmPoNCHn2vCByvYG&oref=https%3A%2F%2Fwww.youtube.com%2Fwatch%3Fv%3DAqCDSq_BXKo%26list%3DPLPC0Udeof3T4NORTjYmPoNCHn2vCByvYG&has_verified=1	https://www.youtube.com/watch?v=oFpddCVIWZg
23	https://www.youtube.com/watch?v=Mc5kqpYyT2c&list=PLPC0Udeof3T4NORTjYmPoNCHn2vCByvYG	https://www.youtube.com/watch?v=HRyvXEsHMts
24	https://www.youtube.com/watch?v=5iSZIbBnksc&list=PLPC0Udeof3T4NORTjYmPoNCHn2vCByvYG&oref=https%3A%2F%2Fwww.youtube.com%2Fwatch%3Fv%3D5iSZIbBnksc%26list%3DPLPC0Udeof3T4NORTjYmPoNCHn2vCByvYG&has_verified=1	https://www.youtube.com/watch?v=9ic_X1KllpQ
25	https://www.youtube.com/watch?v=Zr5HQTfKsvY&list=PLPC0Udeof3T4NORTjYmPoNCHn2vCByvYG	https://www.youtube.com/watch?v=kdCQYA3YHiw
26	https://www.youtube.com/watch?v=YVTZSLUtLOQ&list=PLPC0Udeof3T4NORTjYmPoNCHn2vCByvYG&oref=https%3A%2F%2Fwww.youtube.com%2Fwatch%3Fv%3DYVTZSLUtLOQ%26list%3DPLPC0Udeof3T4NORTjYmPoNCHn2vCByvYG&has_verified=1	https://www.youtube.com/watch?v=HPobgojey3U

27	https://www.youtube.com/watch?v=1dCRW9bfa7o&list=PLPC0Udeof3T4NORTjYmPoNCHn2vCByvYG	https://www.youtube.com/watch?v=RqH4YJtDZuQ
28	https://www.youtube.com/watch?v=fSvm8mDZyOY&list=PLPC0Udeof3T4NORTjYmPoNCHn2vCByvYG	https://www.youtube.com/watch?v=2SI-ZGf91Wc
29	https://www.youtube.com/watch?v=dcHWSKMD96w&list=PLPC0Udeof3T4NORTjYmPoNCHn2vCByvYG	https://www.youtube.com/watch?v=kPGm_u85Qm8
30	https://www.youtube.com/watch?v=pQAewAts0kc&list=PLPC0Udeof3T4NORTjYmPoNCHn2vCByvYG	https://www.youtube.com/watch?v=YMH2xZoQdhw
31	https://www.youtube.com/watch?v=hfN8cHOHACY&list=PLPC0Udeof3T4NORTjYmPoNCHn2vCByvYG	https://www.youtube.com/watch?v=a0eqVMTUkIc
32	https://www.youtube.com/watch?v=q4KggDHXDfU&list=PLPC0Udeof3T4NORTjYmPoNCHn2vCByvYG	https://www.youtube.com/watch?v=isSjcKMHxU4
33	https://www.youtube.com/watch?v=wISKzpsGqI8&list=PLPC0Udeof3T4NORTjYmPoNCHn2vCByvYG	https://www.youtube.com/watch?v=8qQwTo_9Cic
34	https://www.youtube.com/watch?v=GJmz9wP3HWk&list=PLPC0Udeof3T4NORTjYmPoNCHn2vCByvYG	https://www.youtube.com/watch?v=eHQ1K_sTLpo
35	https://www.youtube.com/watch?v=_Er7GGQ6l1g&list=PLPC0Udeof3T4NORTjYmPoNCHn2vCByvYG	https://www.youtube.com/watch?v=lC3hDrR8eSM
36	https://www.youtube.com/watch?v=HxSVEK3Wtp8&list=PLPC0Udeof3T4NORTjYmPoNCHn2vCByvYG	https://www.youtube.com/watch?v=9wHnUHrtAkc
37	https://www.youtube.com/watch?v=zaJBBOnucXA&list=PLPC0Udeof3T4NORTjYmPoNCHn2vCByvYG	https://www.youtube.com/watch?v=ZmKPDQcV0hw
38	https://www.youtube.com/watch?v=yDigYxTQx68&list=PLPC0Udeof3T4NORTjYmPoNCHn2vCByvYG	https://www.youtube.com/watch?v=Hn-xzBUCRLk
39	https://www.youtube.com/watch?v=gzW5tKCXrqg&list=PLPC0Udeof3T4NORTjYmPoNCHn2vCByvYG	https://www.youtube.com/watch?v=S4HBBlGYqSc
40	https://www.youtube.com/watch?v=Ude2OMpyX6g&list=PLPC0Udeof3T4NORTjYmPoNCHn2vCByvYG	https://www.youtube.com/watch?v=sZy6l_DPKoQ
41	https://www.youtube.com/watch?v=e5Ss14Q0MwY&list=PLPC0Udeof3T4NORTjYmPoNCHn2vCByvYG	https://www.youtube.com/watch?v=MLiV_4lVyl0
42	https://www.youtube.com/watch?v=Fl0RyY1l3zU&list=PLPC0Udeof3T4NORTjYmPoNCHn2vCByvYG	https://www.youtube.com/watch?v=3qW_kfvoJws
43	https://www.youtube.com/watch?v=aBFLhDlscvM&list=PLPC0Udeof3T4NORTjYmPoNCHn2vCByvYG	https://www.youtube.com/watch?v=GXm402nGQa0
44	https://www.youtube.com/watch?v=8Vocsknjnco&list=PLPC0Udeof3T4NORTjYmPoNCHn2vCByvYG	https://www.youtube.com/watch?v=cIqnXzqaOfA
45	https://www.youtube.com/watch?v=vWrlnU6Fp28&list=PLPC0Udeof3T4NORTjYmPoNCHn2vCByvYG	https://www.youtube.com/watch?v=0ZJ3P2RXHlw
46	https://www.youtube.com/watch?v=nOxOfNjV3iQ&list=PLPC0Udeof3T4NORTjYmPoNCHn2vCByvYG	https://www.youtube.com/watch?v=ZPSJdOLQyRU
47	https://www.youtube.com/watch?v=ORyxfZnE7I8&list=PLPC0Udeof3T4NORTjYmPoNCHn2vCByvYG	https://www.youtube.com/watch?v=Fh-FeEHpf94
48	https://www.youtube.com/watch?v=l10j1aMe50A&list=PLPC0Udeof3T4NORTjYmPoNCHn2vCByvYG	https://www.youtube.com/watch?v=NqCmN42FbDI
49	https://www.youtube.com/watch?v=gnZYrQiYXYw&list=PLPC0Udeof3T4NORTjYmPoNCHn2vCByvYG	https://www.youtube.com/watch?v=99EtGK3E-Ik
50	https://www.youtube.com/watch?v=d1dYBeQSiEc&list=PLPC0Udeof3T4NORTjYmPoNCHn2vCByvYG	https://www.youtube.com/watch?v=GknjLfJeGSM
51	https://www.youtube.com/watch?v=Iw1H64mFnrU	https://www.youtube.com/watch?v=zRR08wVg-PY
