# Supplementary material for: Social media video analysis methodology for sarin exposure
Source: Forensic Sci Res. 2020 Nov 5;7(2):279–84. doi: 10.1080/20961790.2020.1825061 (PMC9246001; doi:10.1080/20961790.2020.1825061)
Supplement: Supplemental Material [file TFSR_A_1825061_SM6633.zip › the questionnaire.pdf]

The Video Score Questionnaire

Full name: Video no: Date:

No	Symptoms	Present	Absent
1.	Coma		
2.	Loss of consciousness/Unconsciousness		
3.	Wonder/confusion		
4.	Loss of muscle tone		
5.	Muscle weakness (Flaccid or spastic muscle paralysis)		
6.	Muscle contraction and withdrawals		
7.	Convulsion		
8.	Giddiness		
9.	Headache		
10.	Diaphoresis		
11.	Lacrimation		
12.	Excessive salivation		
13.	Cough		
14.	Bronchospasm		
15.	Dispnea		
16.	Respiratory failure /arrest		
17.	Circulatory failure		
18.	Nausea/ Vomiting		
19.	Decontamination		
20.	O_2 therapy		

Instructions

1. Please provide full name, video number and date prior to filling in the data.
2. Once a video ends, please indicate the symptoms immediately.
3. Do not hesitate to review a video multiple times as necessary.
